# The epidemiology of chronic kidney disease and the association with non-communicable and communicable disorders in a population of sub-Saharan Africa

**DOI:** 10.1371/journal.pone.0205326

**Published:** 2018-10-31

**Authors:** Nikolai C. Hodel, Ali Hamad, Claudia Praehauser, Grace Mwangoka, Irene Mndala Kasella, Klaus Reither, Salim Abdulla, Christoph F. R. Hatz, Michael Mayr

**Affiliations:** 1 Medical Outpatient Department, University Hospital Basel, Basel, Switzerland; 2 Swiss Tropical and Public Health Institute, Basel, Switzerland; 3 Ifakara Health Institute, Dar es Salaam/Bagamoyo, Tanzania; 4 Bagamoyo District Hospital, Bagamoyo, Tanzania; 5 University of Basel, Basel, Switzerland; Istituto Di Ricerche Farmacologiche Mario Negri, ITALY

## Abstract

In sub-Saharan Africa (SSA), epidemiological data for chronic kidney disease (CKD) are scarce. We conducted a prospective cross-sectional study including 952 patients in an outpatient clinic in Tanzania to explore CKD prevalence estimates and the association with cardiovascular and infectious disorders. According to KDIGO, we measured albumin-to-creatinine ratio and calculated eGFR using CKD-EPI formula. Factors associated with CKD were calculated by logistic regression. Venn diagrams were modelled to visualize interaction between associated factors and CKD. Overall, the estimated CKD prevalence was 13.6% (95% CI 11–16%). Ninety-eight patients (11.2%) (95% CI 9–14%) were categorized as moderate, 12 (1.4%) (95% CI 0–4%) as high, and 9 (1%) (95% CI 0–3%) as very high risk according to KDIGO. History of tuberculosis (OR 3.75, 95% CI 1.66–8.18; p = 0.001) and schistosomiasis (OR 2.49, 95% CI 1.13–5.18; p = 0.02) were associated with CKD. A trend was seen for increasing systolic blood pressure (OR 1.02 per 1 mmHg, 95% CI 1.00–1.03; p = 0.01). Increasing BMI (OR 0.92 per 1kg/m^2^, 95% CI 0.88–0.96; p = <0.001) and haemoglobin (OR 0.82 per 1g/dL, 95% CI 0.72–0.94; p = 0.004) were associated with risk reduction. Diabetes was associated with albuminuria (OR 2.81, 95% CI 1.26–6.00; p = 0.009). In 85% of all CKD cases at least one of the four most common factors (hypertension, diabetes, anaemia, and history of tuberculosis or schistosomiasis) was associated with CKD. A singular associated factor was found in 61%, two in 14%, and ≥3 in 10% of all CKD cases. We observed a high prevalence estimate for CKD and found that both classical cardiovascular and neglected infectious diseases might be associated with CKD in a semi-rural population of SSA. Our finding provides further evidence for the hypothesis that the “double burden” of non-communicable and endemic infectious diseases might affect kidney health in SSA.

## Introduction

Chronic kidney disease (CKD) is increasingly recognized as a global public health problem with major impact on health, health-care costs and productivity [1, 2]. However, epidemiological data in developing countries are still scarce or of limited quality [3, 4]. There is a strong interaction between cardiovascular risk factors and CKD, whereby diabetes and hypertension confer the highest risk for developing CKD [1, 2]. Conservative projections for developing countries and regions including sub-Saharan Africa (SSA), expect a dramatic increase in diabetes, hypertension, and obesity for the coming decade [5–9], which raises fears of a sharp increase in CKD [2, 10, 11]. Additionally, in SSA the expected epidemic of cardiovascular diseases strikes populations, which already suffer from a high burden of communicable diseases [12–14].

However, up to now, the impact of the double burden of communicable and non-communicable diseases on the development of CKD has been poorly studied [3, 15]. In a recently published systematic review problems and weaknesses of existing CKD prevalence and risk factor studies in SSA are discussed [8]. One difficulty is the lack of reliable and validated measurements of kidney function [8]. The CKD-EPI formula, which is thought to most closely approximate glomerular filtration rate (GFR) in African populations, was only used in a very small number of studies [8, 16, 17]. Further, measurements of proteinuria, beside GFR the most important marker of CKD, were not routinely performed or done only semi-quantitatively with limitations in sensitivity and specificity [8]. Finally, in SSA there is a need to explore the association of both infectious and non-communicable risk factors with CKD [8].

Treatment of early CKD can slow or prevent progression to end-stage renal disease (ESRD) and reduce cardiovascular mortality [2, 18, 19]. Epidemiological data are of great importance to know most exactly what the magnitude of the problem is, what risk factors are, and how screening and treatment programs might look like in afflicted areas. The aim of our study was to provide high quality data on CKD prevalence estimates according to KDIGO stages and to analyse the association with both, classical cardiovascular risk factors and endemic communicable diseases in a region of SSA. Because most published studies were conducted in urban and/or rural populations [20–26], we performed our study in a semi-rural region. For this, the semi-rural district of Bagamoyo in Tanzania qualified to complete the currently available data set for CKD prevalence estimates in SSA.

## Material and methods

### Study population and setting

We conducted a single centre cross-sectional study at the outpatient clinic (OPC) of the Bagamoyo District Hospital (BDH) in Tanzania. The BDH is located in the Bagamoyo township on the coast of the Indian Ocean, 65 km north of Dar es Salaam. The hospital provides care for a semi-rural population of the Bagamoyo district in the Pwani region, with a total population of about 300`000 inhabitants counted in the 2012 nationwide census [27]. The national sex ratio was 51% females to 49% males and the population growth rate was 2.7% [27]. In the BDH the most frequent disorders for adults were malaria, hypertension, heart diseases and infectious diseases such as TB, pneumonia and HIV/Aids. However, precise epidemiological data on disease prevalence are lacking. The outpatient clinic (OPC) were visited on average by 120 (range 41–164) patients daily (survey performed from NH from 01.12.2010 to 31.05.2011). To ensure a highly standardized procedure one consultation hour of the general outpatient ward was designated for the current study. The consultation hour was led by one local medical officer (IMK) and two local nurses. From the newly registered patients of the OPC consecutively 15 to 20 patients per day were seen in the designated consultation hour. The call up of the patients from the OPC ward was done by the local medical staff without any involvement of the investigators.

After informed consent, all patients ≥ 18 years, irrespective of the reason of consultation, were included. Pregnant women and patients who were not able or willing to provide an informed signed consent were excluded. The study (RenalOne study) was performed from 08.12.2010 to 30.05.2011.

### Measurements and procedures

All data were collected in a case report form, translated from English to Swahili. In all participants body weight and height, blood pressure, heart rate and temperature were recorded. After informed consent, a blood sample was taken for complete blood count and serum creatinine. Complete blood count was performed by a Sysmex Xs 800i analyser. Serum creatinine was measured using Creatinine Jaffe Gen2 reagent on a Cobas Integra 400 plus analyser and calibrated to IDMS standards by the Ifakara Health Institute (IHI) laboratory at the Bagamoyo site center. HbA1c was measured from capillary blood by using a bed-side DCA 2000+ Analyzer (Siemens Healthcare Diagnostics). After informed consent, HIV-screening was done with an immunochromatographic test for antibodies to HIV-1 and HIV-2 (test kits: Uni-Gold HIV, Trinity Biotech, Ireland; Determine HIV-1/2, Inverness Medical Japan, Japan; SD BIOLINE HIV-1/2 3.0, SD Standard Diagnostics, Korea). All participants were instructed to void a clean-urine specimen. Urine samples were prepared for microscopic analysis. Albumin-to-creatinine ratio (ACR) was measured using a DCA 2000+ analyser (Siemens Healthcare Diagnostics) [28].

CKD was defined as the presence of either impaired kidney function and/or albuminuria based on a one-time measurement. When one-time measurements are used, prevalence of reduced GFR and albuminuria might be overestimated due to physiological variation and temporarily elevated values after physical activity and during acute illness or dehydration [29]. Nevertheless, one-time measurements have been used for screening and epidemiologic purposes, as longitudinal documentation is not usually available in epidemiological studies [30–32]. To minimize the risk of overestimation CKD rates patients with suspicion of acute systemic infection/inflammation or possible urinary tract infection (UTI) were excluded from the calculation of CKD prevalence rates, i.e. all patients with body temperature of ≥ 38.5°C (armpit), acute malaria, acute TB, or leukocyte count >20/HPF in urinary sediment. Kidney function was assessed by eGFR using the CKD-EPI formula [28, 33]. CKD was defined as an eGFR of <60 ml/min/1.73m^2^ and/or an ACR of ≥30mg/g (≥3 mg/mmol) and categorized according to KDIGO risk stages [28, 34]. The KDIGO risk stages (low, moderate, high and very high risk) describe the relative risks of major complications of CKD such as all-cause mortality, cardiovascular mortality, kidney failure (ESRD), AKI and progression of CKD. The categories are based on meta-analysis (adjusted relative risk) for general population cohorts with ACR or dipstick within different stages of kidney function (eGFR). Patients with an adjusted relative risk from 1–8, 9–14, 15–21, and 22–28, were ranked as low, moderate, high, and very high risk for the above mentioned complications, respectively [28, 34, 35].

Office blood pressure (BP) was assessed by a single measurement using a manual sphygmomanometer in a sitting position after 5 minutes at rest. BP was regarded as “elevated BP” if BP was ≥140/90 mmHg. Anaemia was defined as Hb <13.0 g/dl in male and <12.0 g/dl in female patients [36]. Diabetes mellitus was defined as a history of diabetes, the use of antidiabetic medication or a HbA1c of ≥ 6.5% [37, 38]. Diagnosis of malaria was based on a positive immunochromatographic test (SD BIOLINE Malaria Antigen P.f/Pan, SD Standard Diagnostics, Korea).

### Outcomes

Primary outcome of the study were prevalence estimates of CKD risk groups and the association of non-communicable and communicable disorders with CKD. Secondary outcome was the frequency and the magnitude of the overlap between the most frequent associated factors in patients with CKD and vice versa.

### Ethical considerations

All participants signed an informed consent form in Swahili. For illiterate patients, the informed consent was read, and fingerprint of the index finger was used instead of a signature. The study was approved by the Ethical Committee of the Cantons Basel-Stadt and Basel-Land (University of Basel) in Switzerland (No. 220/10), the Institutional Review Board of the IHI in Tanzania (IHI/IRB/No.20-2010), and approved by the Tanzanian National Institute for Medical Research (NIMR). ClinicalTrials.gov Identiver: NCT03458338.

### Statistical analyses

Statistical analyses were performed using STATA version 14 (StataCorp., College Station, TX, USA) and R (version 3.2.3) [39]. Discrete variables were expressed as counts (percentage), and comparison between groups was done with Pearson’s chi-square test or Fisher’s exact test. Continuous variables were expressed as mean ± standard deviation (SD) if normally distributed or as median and range if not normally distributed, and t-test or Mann-Whitney test were used for comparison between groups. For multinomial outcomes such as CKD risk groups the Sison-Glaz method was used to calculate prevalence estimates and corresponding 95% confidence interval (CI) [40]. Univariate and multivariate logistic regression were used to determine associated factors for CKD. Results were expressed as odds ratio (OR) with 95% CI. P-values of <0.05 were considered as statistically significant. Based on the multivariate regression analysis for CKD, significant factors associated with CKD were chosen for a cross tabulation with CKD. Area-proportional Venn-diagrams were plotted with the online software (http://apps.bioinforx.com) to analyse the relationship between associated factors and CKD [41]. For visualisation in the Venn diagram, the model was restricted to the four most frequent associated factors, whereby tuberculosis (TB) and schistosomiasis were taken together, since both diseases were equally frequent in CKD cases. Furthermore, for the Venn-diagram model elevated BP was defined as BP ≥ 140/90 mmHg.

## Results

**Fig 1:**
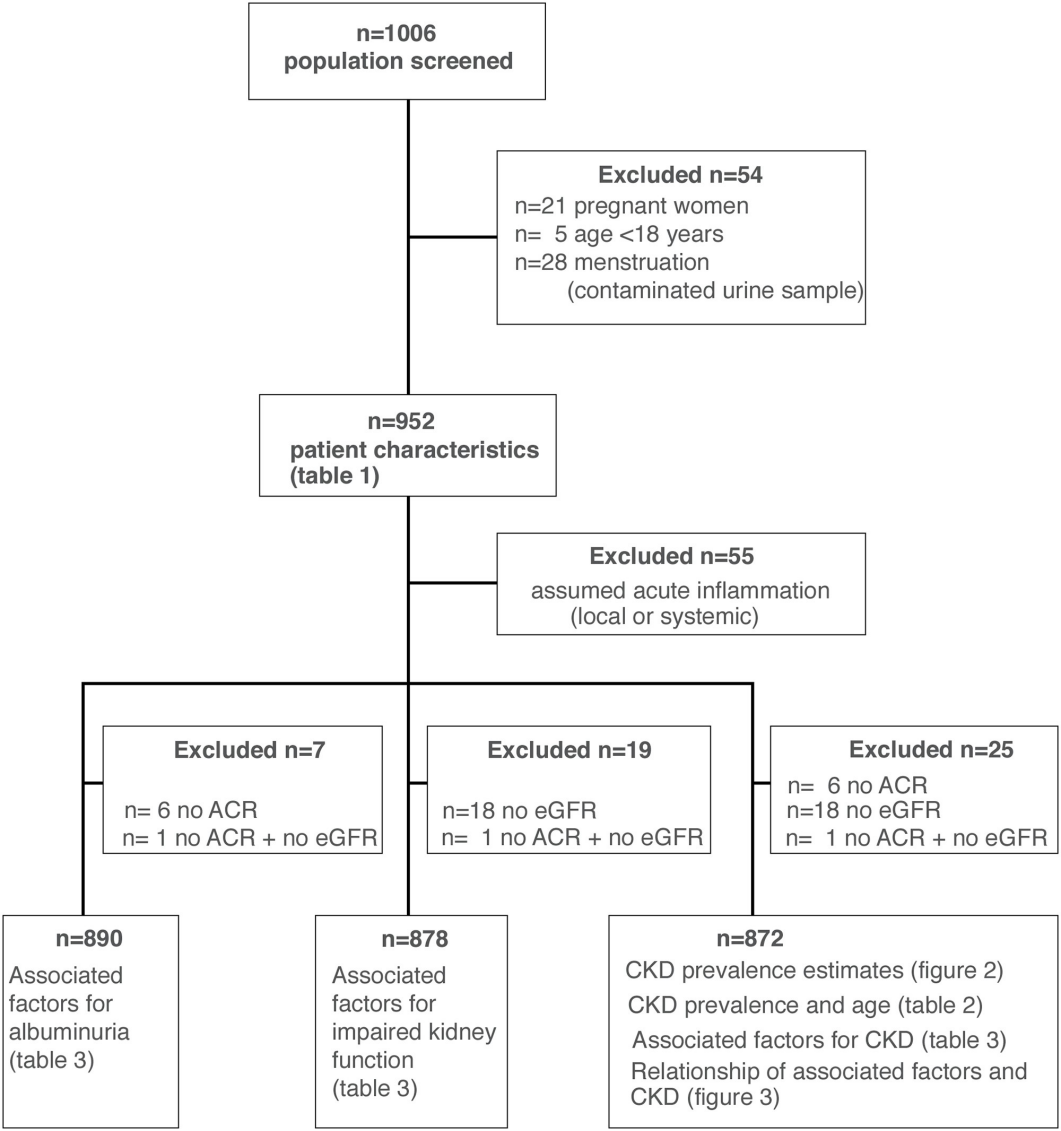
Study flow. ACR: Albumin-creatinine-ratio; eGFR: estimated glomerular filtration rate; albuminuria: ACR ≥30mg/g, (≥3 mg/mmol); impaired kidney function: eGFR <60ml/min/1.73m^2^; CKD: chronic kidney disease (ACR ≥30mg/g (≥3mg/mmol) and/or eGFR <60ml/min/1.73m^2^).

Overall, 1006 patients were recruited (Fig 1). Twenty-one pregnant women and 5 patients aged less than 18 years, and 28 females with urinary samples contaminated by menstruation were excluded, leaving 952 patients for the final analysis. In 55 patients, symptoms and criteria of acute systemic infection/inflammation or possible UTI were seen. These patients were excluded from the calculation of CKD prevalence estimates, the logistic regression analyses, and the Venn diagram (Fig 1).

### Patient characteristics

Patient characteristics are summarized in (Table 1). The study population consisted of 303 (32%) males and 649 (68%) females. Median age was 37 years (range 18–91) and 57% (n = 537) were ≤ 40 years. Median body mass index (BMI) was 25 kg/m^2^ and was significantly higher in women (26 kg/m^2^) than in men (23 kg/m^2^) (p<0.001). Seventy-six (8%) patients had a BMI <18.5 kg/m^2^ and 419 (44%) a BMI ≥ 25 kg/m^2^. Overall, 257 (27%) patients had a BP ≥ 140/90 mmHg, with more female (30%) than male (21%) affected (p = 0.002). More females had a positive history of hypertension (female 19% versus male 10% (p = 0.001)). Anaemia was detected in 312 (33%) patients and the percentage was significantly higher in women (41%) than in men (16%) (p<0.001).

**Table 1:** Patient characteristics.

	n (missing)	Overall	Male	Female	p-value
Overall	952	952	303 (32%)	649 (68%)	
Age (years)	946 (6)	37 [18–91]	36 [18–91]	37 [18–89]	0.59°
BMI (kg/m^2^) ^a^	949 (3)	25 [14–53]	23 [15–41]	26 [14–53]	< 0.001°
BMI<18.5		76 (8%)	23 (8%)	53 (8%)	0.91*
BMI 18.5–24.9		454 (48%)	192 (63%)	262 (41%)	< 0.001*
BMI 25–29.9		226 (24%)	61 (20%)	165 (25%)	0.15*
BMI ≥30		193 (20%)	26 (9%)	167 (26%)	< 0.001*
BP syst mmHg ^b^	949 (3)	124 [70–286]	120 [80–240]	128 [70–286]	0.48°
BP syst <120 mmHg		304 (32%)	86 (28%)	218 (34%)	0.20*
BP syst 120–139 mmHg		302 (32%)	116 (38%)	186 (29%)	0.007*
BP syst 140–159 mmHg		190 (20%)	64 (21%)	126 (20%)	0.79 *
BP syst ≥160 mmHg		153 (16%)	36 (12%)	117 (18%)	0.037*
BP diast mmHg ^c^	949 (3)	80 [36–150]	80 [46–130]	80 [36–150]	0.001°
BP diast <90 mmHg		626 (66%)	223 (74%)	403 (62%)	0.001*
BP diast 90–99 mmHg		142 (15%)	36 (12%)	106 (16%)	0.15 *
BP diast ≥100 mmHg		181 (19%)	43 (14%)	138 (21%)	0.02*
Elevated BP ^d^	949 (3)	257 (27%)	63 (21%)	194 (30%)	0.002*
Serum-creatinine	938 (14)	64 [18–980]	75 [27–480]	59 [18–980]	< 0.001°
eGFR ml/min/1.73m^2^	932 (20)	123 [5–220]	124 [14–218]	123 [5–220]	0.88°
ACR mg/g	945 (7)	7 [1–999]	5 [1–353]	7 [1–999]	< 0.001°
History of hypertension	950 (2)	155 (16%)	31 (10%)	124 (19%)	0.001*
Diabetes mellitus	952	64 (7%)	19 (6%)	45 (7%)	0.78*
Haemoglobin g/dl	879 (73)	12.8 [4.1–22.2]	14.3 [6.3–22.2]	12.2 [4.1–18]	< 0.001°
Anaemia	879 (73)	312 (33%)	43 (16%)	269 (41%)	< 0.001*
Fever ≥ 38.5°C	936 (16)	12 (1%)	4 (1%)	8 (1%)	1.0*
Urinary tract infection ^e^	943(9)	28 (3%)	9 (3%)	19 (3%)	0.1
HIV status unknown	952	230 (24%)	76 (25%)	154 (24%)	0.035*
HIV test negative		658 (69%)	217 (72%)	441 (68%)	0.031*
HIV positive ^f^		64 (7%)	10 (3%)	54 (8%)	0.031*
Malaria acute ^g^	952	18 (2%)	8 (3%)	10 (2%)	0.3*
History of urinary tract infection ^h^	952	10 (1%)	3 (1%)	7 (1%)	0.6*
History of Smoking	952	76 (8%)	67 (22%)	9 (1%)	< 0.001*
History of Schistosomiasis	946 (6)	73 (8%)	45 (15%)	28 (4%)	< 0.001*
History of Malaria	949 (3)	856 (90%)	270 (89%)	586 (90%)	0.49*
History of Tuberculosis	950 (2)	45 (5%)	17 (6%)	28 (4%)	0.41 *

Data are displayed as counts and (percent) or median and [range];

°Mann-Whitney-U (rank sum) test,

*Fisher’s exact test;

^a^BMI: Body mass index (kg/m^2^); underweight (BMI<18.5kg/m^2^), overweight (BMI 25–29.9 kg/m^2^), obesity (BMI ≥30kg/m^2^) were defined according to WHO BMI reference standards [42].

^b^BP syst: Blood pressure systolic,

^c^BP diast: blood pressure diastolic;

^d^Elevated BP: Blood pressure systolic ≥140 and blood pressure diastolic ≥90 mmHg;

^e^Acute urinary tract infection: leucocytes >20 per high power field in microscopy;

^f^HIV positive: 43 patients were diagnosed with HIV by testing within the study, 21 patients had a history of HIV and 15 of them were on antiretroviral therapy;

^g^Acute malaria: positive rapid diagnostic malaria test;

^h^History of urinary tract infection (UTI) > 2 episodes of UTI/year.

Symptoms of acute infection were recorded in 55 (6%) patients, with fever ≥ 38.5°C in 12 (1%), acute malaria in 17 (2%), acute UTI in 24 (3%), and TB in 2 (0.2%) cases. Sixty-four (7%) patients were HIV positive, including 43 (4%) newly diagnosed cases. History of Malaria was reported by 856 (90%) patients, history of schistosomiasis and TB by 73 (8%) and 45 (5%), respectively.

### Prevalence estimates of chronic kidney disease

**Fig 2:**
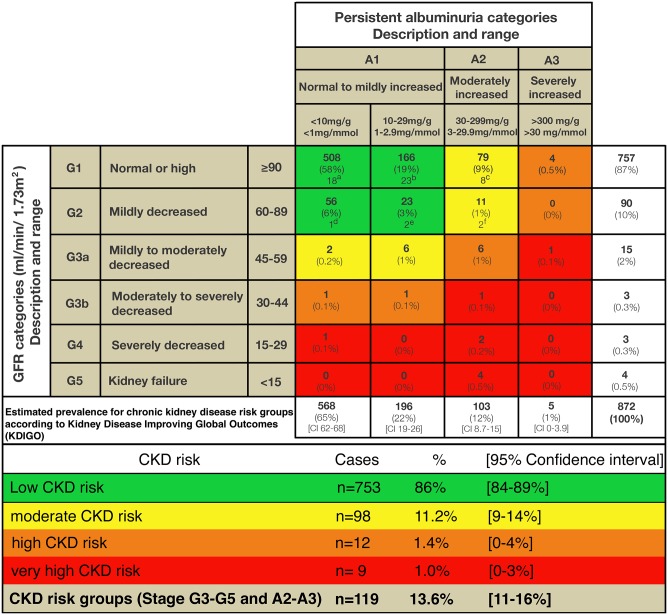
Distribution of patients based on eGFR and albuminuria with prevalence estimates according KDIGO chronic kidney disease risk groups [28]. Green: low CKD risk; yellow: moderate CKD risk; orange: high CKD risk; red: very-high CKD risk. ^a-f^ Subset of patients with evidence of systemic acute infection/inflammation or urinary tract infection (UTI) excluded from prevalence calculation (n = 54; 1/55 with acute infection/inflammation not shown due to missing ACR). ACR: albumin-creatinine-ratio; eGFR: estimated glomerular filtration rate; CKD: chronic kidney disease; KDIGO: Kidney disease improving global outcomes.

The overall estimated prevalence of CKD (eGFR categories G3a-G5 and albuminuria categories A2-A3) was 13.6% (n = 119) (95% CI 11–16%). Ninety-eight patients (11.2%) (95% CI 9–14%) were assigned to the moderate, 12 (1.4%) (95% CI 0–4%) to the high, and 9 (1%) (95% CI 0–3%) to the very high CKD risk group according to KDIGO (Fig 2). Seven hundred-fifty-three patients (86.4%) (95% CI 84–89%) had an eGFR ≥60ml/min/1.73m^2^ and an ACR <30 mg/g (3mg/mmol) and therefore, in the absence of other markers of kidney injury, a low-risk prognosis (Fig 2).

### Prevalence estimates for chronic kidney disease across age groups

The distribution of albuminuria, reduced eGFR, and CKD, are depicted in Table 2. There was a clear increase in the rate of reduced eGFR and CKD across the age groups with most cases in patients equal to or older than 65 years (p<0.001). Albuminuria was most often seen in patients 40–64 years old (p<0.001) (Table 2).

**Table 2:** Prevalence estimates for chronic kidney disease across age groups.

Age group	18–39 years	40–64 years	≥65 years	Total
	n	n	n	n
Total	459	334	79	872
Chronic kidney disease (CKD)	40 (8.7%)	51 (15.3%)	28 (35.4%)	119
eGFR^a^ <60ml/min/1.73m^2^	4 (0.8%)	11 (3.3%)	10 (12.7%)	25
ACR^b^ > 30mg/g	39 (8.5%)	48 (14.4%)	21 (6.3%)	108

^a^eGFR: estimated glomerular filtrations rate.

^b^ACR: albumin-creatinine-ratio.

### Factors associated with albuminuria

In univariate logistic regression older age (OR 1.03 per 1year, 95%CI 1.02–1.04; p<0.001), higher systolic BP levels (OR 1.02 per 1mmHg, 95%CI 1.01–1.03; p<0.001), higher diastolic BP levels (OR 1.03 per 1mmHg, 95%CI 1.02–1.05; p<0.001), a history of hypertension (OR 2.13, 95%CI 1.33–3.34; p<0.001), diabetes mellitus (OR 3.06, 95%CI 1.64–5.48; p<0.001), and a history of TB (OR 3.28, 95%CI 1.56–6.52; p = 0.001) were associated with albuminuria. Increasing haemoglobin levels were associated with a risk reduction (OR 0.88 per 1g/dL, 95%CI 0.80–0.98) (Table 3).

**Table 3:** Factors associated with albuminuria, impaired kidney function, and chronic kidney disease.

Associated factors	Factors associated with albuminuria				Factors associated with impaired kidney function				Factors associated with chronic kidney disease			
	Univariate		Multivariate		Univariate		Multivariate		Univariate		Multivariate	
	OR (CI)	p-value	OR (CI)	p-value	OR (CI)	p-value	OR (CI)	p-value	OR (CI)	p-value	OR (CI)	p-value
Age (years)	1.03 (1.02–1.04)	<0.001	1.00 (0.99–1.02)	0.68	1.06 (1.04–1.09)	<0.001	1.05 (1.01–1.09)	0.007	1.04 (1.02–1.05)	<0.001	1.01 (0.99–1.03)	0.18
Sex (female vs. male)	0.90 (0.59–1.38)	0.62	0.64 (0.35–1.19)	0.16	0.82 (0.36–1.96)	0.64	0.22 (0.06–0.75)	0.01	0.86 (0.58–1.31)	0.48	0.63 (0.35–1.15)	0.13
BMI (kg/m^2^)^a^	0.98 (0.95–1.02)	0.33	0.93 (0.88–0.97)	0.002	1.03 (0.97–1.10)	0.28	0.98 (0.89–1.06)	0.60	0.98 (0.95–1.02)	0.32	0.92 (0.88–0.96)	<0.001
BP syst (mmHg)^b^	1.02 (1.01–1.03)	<0.001	1.01 (1.00–1.03)	0.02	1.02 (1.01–1.03)	0.002	1.00 (0.97–1.02)	0.95	1.02 (1.01–1.03)	<0.001	1.02 (1.00–1.03)	0.01
BP diast (mmHg)^c^	1.03 (1.02–1.05)	<0.001	1.02 (0.99–1.04)	0.16	1.04 (1.02–1.06)	<0.001	1.03 (0.98–1.08)	0.21	1.03 (1.02–1.05)	<0.001	1.01 (0.99–1.03)	0.25
History of hypertension	2.13 (1.33–3.34)	<0.001	1.29 (0.68–2.40)	0.42	4.06 (1.77–9.11)	<0.001	2.74 (0.78–9.33)	0.11	2.45 (1.57–3.78)	<0.001	1.50 (0.82–2.72)	0.18
Diabetes mellitus	3.06 (1.64–5.48)	<0.001	2.81 (1.26–6.00)	0.009	1.87 (0.43–5.60)	0.32	0.32 (0.02–2.01)	0.32	3.22 (1.77–5.58)	<0.001	2.20 (0.98–4.71)	0.05
Haemoglobin (g/dl)	0.88 (0.80–0.98)	0.02	0.82 (0.72–0.94)	0.005	0.78 (0.64–0.95)	0.01	0.62 (0.46–0.82)	<0.001	0.88 (0.80–0.98)	0.02	0.82 (0.72–0.94)	0.004
HIV positive vs. negative^d^	1.24 (0.53–2.59)	0.59	0.77 (0.28–1.87)	0.58	0.65 (0.04–3.28)	0.68	0.25 (0.01–2.09)	0.29	1.08 (0.46–2.25)	0.85	0.65 (0.23–1.57)	0.37
HIV negative vs. unknown	1.06 (0.65–1.68)	0.81	0.62 (0.31–1.17)	0.16	1.24 (0.47–2.92)	0.64	0.24 (0.03–1.05)	0.10	0.90 (0.55–1.43)	0.88	0.52 (0.26–0.99)	0.06
History of UTI^e^	0.79 (0.04–4.29)	0.83	2.29 (0.12–13.79)	0.45	4.00 (0.21–22.66)	0.20	16.9 (0.63–200.7)	0.04	0.70 (0.04–3.85)	0.75	2.14 (0.11–12.9)	0.49
History of smoking	1.01(0.46–2.00)	0.97	1.19 (0.44–2.89)	0.71	0.47 (0.03–2.26)	0.46	0.67 (0.03–4.64)	0.72	0.95 (0.43–1.87)	0.84	0.98 (0.36–2.36)	0.96
History of tuberculosis	3.28 (1.56–6.52)	0.001	3.80 (1.65–8.36)	0.001	4.31 (1.21–12.06)	0.01	5.53 (1.32–19.5)	0.01	3.29 (1.60–6.46)	<0.001	3.75 (1.66–8.18)	0.001
History of schistosomiasis	1.62 (0.80–3.04)	0.15	2.28 (1.01–4.79)	0.04	1.73(0.40–5.17)	0.38	4.34 (0.81–18.07)	0.06	1.66 (0.84–3.07)	0.12	2.49 (1.13–5.18)	0.02

Factors associated with albuminuria: ACR ≥30mg/g, (≥3 mg/mmol); impaired kidney function: eGFR <60ml/min/1.73m^2^; chronic kidney disease: eGFR <60ml/min/1.73m^2^ and/orACR ≥30 mg/g (≥3mg/mmol);

^a^BMI: Body mass index (kg/m^2^);

^b^BP syst: Blood pressure systolic;

^c^BP diast: blood pressure diastolic,

^d^HIV positive: 43 patients were diagnosed within the study, 21 patients had a history of HIV and 15 of them were on antiretroviral therapy.

^e^History of urinary tract infection (UTI): >2 episodes of UTI/year.

ACR: albumin-creatinine-ration; eGFR: estimated glomerular filtration rate. All variables shown in the table were included in the multivariate logistic regression analysis.

In multivariate logistic regression analysis, diagnosis of diabetes mellitus (DM) (OR 2.81, 95%CI 1.26–6.00; p = 0.009), a history of TB (OR 3.80, 95%CI 1.65–8.36; p = 0.001), and a history of schistosomiasis (OR 2.28, 95%CI 1.01–4.79; p = 0.04) were associated factors for albuminuria (Table 2). A strong trend was seen for increasing systolic BP levels (OR 1.01 per 1mmHg, 95%CI 1.00–1.03, p = 0.02). An increase in haemoglobin (OR 0.82 per 1g/dL, 95%CI 0.72–0.94 p = 0.005) and an increasing BMI (OR 0.93 per 1kg/m^2^, 95%CI 0.88–0.97; p = 0.002) were associated with risk reduction. (Table 3).

### Factors associated with impaired kidney function

In univariate logistic regression older age (OR 1.09 per 1year, 95%CI 1.04–1.09; p<0.001), increasing systolic BP (OR 1.02 per 1mmHg, 95%CI 1.01–1.03; p = 0.002), diastolic BP levels (OR 1.04 per 1mmHg, 95%CI 1.02–1.06; p<0.001), a history of hypertension (OR 4.06, 95%CI 1.77–9.11; p<0.001), and history of TB (OR 4.31, 95%CI 1.21–12.06; p = 0.01) were associated with impaired kidney function. Increasing haemoglobin levels were associated with risk reduction (OR 0.78 per 1g/dL, 95%CI 0.64–0.95; p = 0.01) (Table 3).

In multivariate logistic regression analysis, older age (OR 1.05 per 1year, 95%CI 1.01–1.09; p = 0.007) and a history of TB (OR 5.53, 95%CI 1.32–19.5; p = 0.01) were associated with impaired kidney function (Table 2). Female sex (OR 0.22, 95%CI 0.06–0.75; p = 0.01) and increasing haemoglobin levels were associated with risk reduction (OR 0.62 per 1g/dL, 95%CI 0.46–0.82; p<0.001). (Table 3).

### Factors associated with chronic kidney disease

In univariate logistic regression older age (OR 1.04, per 1year 95%CI 1.02–1.05; p<0.001), increasing systolic BP (OR 1.02 per 1mmHg, 95%CI 1.01–1.03, p<0.001), increasing diastolic BP (OR 1.03 per 1mmHg, 95%CI 1.02–1.05, p<0.001), a history of hypertension (OR 2.45, 95%CI 1.57–3.78; p<0.001), diabetes mellitus (OR 3.22, 95%CI 1.77–5.58, p<0.001), and a history of tuberculosis (OR 3.29, 95%CI 1.60–6.46; p<0.001) were associated with CKD (Table 3).

In multivariate logistic regression analysis, a history of TB (OR 3.75, 95%CI 1.66–8.18; p = 0.001), and history of schistosomiasis (OR 2.49, 95%CI 1.13–5.18; p = 0.02) were associated with CKD (Table 3). A strong trend was seen for increasing systolic BP levels (OR 1.02 per 1mmHg, 95%CI 1.00–1.03; p = 0.01). An increasing BMI (OR 0.92 per 1kg/m^2^, 95%CI 0.88–0.96; p<0.001) and increasing haemoglobin levels were associated with risk reduction (OR 0.82 per 1g/dL, 95%CI 0.72–0.94; p = 0.004) (Table 3).

### Relationship of chronic kidneys disease and associated factors

**Fig 3:**
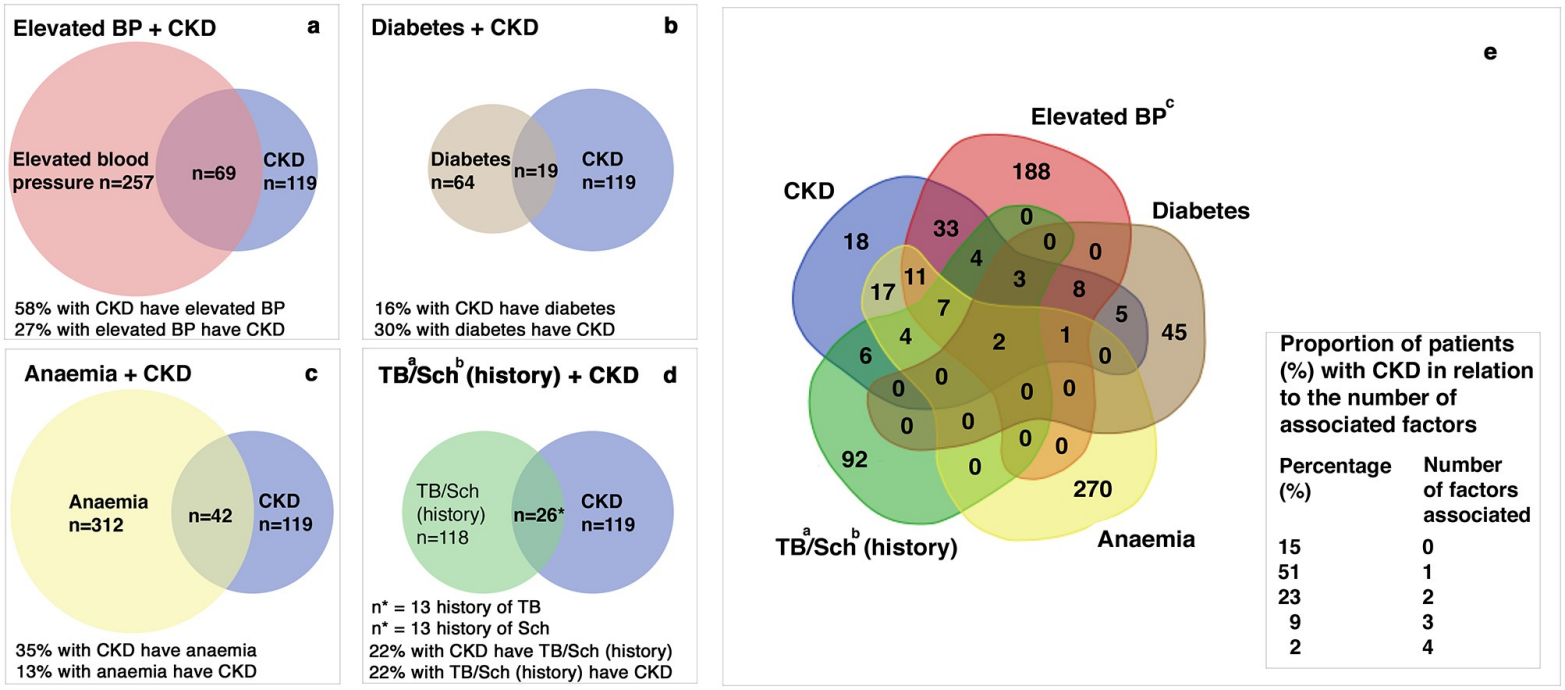
Venn diagram for interaction between chronic kidney disease and associated factors. (a-d) Relationship between associated factors and chronic kidney disease (CKD). Overlapping areas: patients with CKD and a single associated factor and vice versa; (e) Interaction between the four associated factors,—elevated BP (red), diabetes (brown), anaemia (yellow) and history of schistosomiasis/tuberculosis (green) -, in relation to CKD (blue). The numbers indicate patients with CKD, one or more associated factors and overlap between CKD and associated factors. Of all CKD cases 85% (n = 101) are overlapping at least one associated factor, 15% (n = 18) are not associated to one of these factors. ^a^ TB: tuberculosis ^b^Sch: schistosomiasis; ^c^Elevated BP: elevated blood pressure (≥140/90 mmHg); CKD: chronic kidney disease (eGFR <60ml/min/1.73m2 or albumin-to-creatinine ratio (ACR) ≥30mg/g (≥3mg/mmol)).

The relationship of associated factors and CKD and vice versa is depicted in Fig 3. In 85% (n = 101/119) of all CKD cases at least one of the following four associated factors, elevated BP, anaemia, diabetes, or a history of TB or schistosomiasis, was present. A singular associated factor was seen in 61% (n = 72/119) CKD cases, predominantly elevated BP in 28% and anaemia in 24% of the cases. Two associated factors were found in 14% (n = 17/119) and three or more associated factors in 10% (n = 12/119) of all CKD cases. A history of TB (n = 13) or schistosomiasis (n = 13) was found in 22% of all CKD cases (Fig 3).

## Discussion

The main finding of our study was a high overall prevalence estimate (13.6%) for CKD. Interestingly, we found an association of both classical cardiovascular disorders and endemic infectious diseases with CKD. Reports from epidemiological studies pointed out, that the “double burden” could account for an increased CKD prevalence in populations from SSA [21, 22, 24]. People living in low- and middle-income countries such as Tanzania are especially vulnerable, because non-communicable diseases are on a steady rise. This trend is driven by epidemiologic transition, population aging, rapid urbanization processes and lifestyle changes in the region [3, 7, 12, 22, 43, 44]. In the same time in which the prevalence of cardiovascular disorders increases endemic infectious diseases such as TB, HIV/Aids, Malaria and Schistosomiasis remain still highly prevalent [3, 12, 45, 46]. Our findings provide further evidence for the hypothesis that the “double burden” of non-communicable and endemic infectious diseases might affect kidney health in SSA [21, 22, 47].

At least one of the following factors,—elevated BP, diabetes, anaemia, or a history of TB or schistosomiasis—, was present in most of all CKD cases. In contrast, in two similar studies 50% [22] and 80% [21] of all CKD cases could not be attributed to the explored risk factors, which have been hypertension, diabetes and HIV [21, 22]. This discrepancy emphasizes the importance to include endemic neglected diseases to explore their role in CKD.

Due to the large number of unexplained CKD cases, Peck et al hypothesized, that schistosomiasis could be an important cause of CKD in SSA [21]. A history of schistosomiasis was self-reported by 8% with a significantly different distribution between male and female. The estimated schistosomiasis prevalence for the year 2010 in Tanzania was 53.3% with the second highest prevalence in SSA after Nigeria [45, 48]. At first glance, these data are very different from our finding, but there are known regional differences in prevalence and intensity of Schistosoma infection in Tanzania [49]. Additionally, estimated prevalence rates of schistosomiasis were projected from the number of people treated in 2009 [48, 50]. It was estimated that 1.3 million infected people were treated, which means a national treatment coverage rate of only 3.6% [48]. In our survey, we can assume that most of the people who confirmed schistosomiasis have been treated and therefore could remember on schistosomiasis. Thus, the prevalence rate of 8% mirrors probably rather the treatment rate than the true prevalence rate of schistosomiasis. Interestingly, in our study a history of schistosomiasis was a significant factor associated with albuminuria and CKD. A recent hospital-based study in HIV positive children from Mwanza Tanzania has shown that schistosomiasis was the only factor that was statistically significant (OR 2.51, 95% CI 1.46–4.31) associated with renal dysfunction [51]. In spite of the weaknesses of self-reported data, our results indicate a similar risk for CKD and support the hypothesis that Schistosoma infection could be an important risk factor for CKD, at least in regions with high prevalence as in Tanzania [51].

A history of TB was recorded in 5% of the patients. The cases reported were distributed over a long period of time, i.e. from 1962 until the year of enrolment. However, a quarter of all reported TB cases (24.4%) has occurred within the past three years. This unequal distribution across the time might be a consequence of recall bias or of an increased awareness of TB in recent years [46, 52]. Our study provides further evidence that TB is a factor associated for CKD as recently summarized [53]. However, little is known about the underlying mechanism and therefore the causal role of TB for CKD has further to be explored.

As seen in other studies, elevated BP and diabetes were also very common in our population [22, 54, 55]. Both are well known risk factors for albuminuria and reduced kidney function. On the other hand, obesity is a known risk factor for diabetes, hypertension and has therefore major impact on cardiovascular diseases [56]. On contrast to this, our data showed that an increasing BMI might have a protective role in CKD. This contradictory result could be explained by the presence of underweight in a part of our population. In developing countries, a higher BMI might be a surrogate for better health and living conditions, and therefore acts to a certain degree as a protection factor for CKD [20–22]. However, in line with the prevalence trend in industrialized countries, overweight and obesity are increasing in the developing world [6] and populations are exposed to both extremes, underweight and obesity, both known to increase the risk for infectious diseases and cardiovascular disorders, and therefore also for CKD [57].

As far as we know, this is the first study, which included anaemia as a factor associated with CKD in SSA [21, 22, 58–60]. Anaemia was highly prevalent, strongly connected to CKD and associated with albuminuria and impaired kidney function. Although, the cause of anaemia was not in the focus of the study, the predominance in females indicates that childbirths and menstruation are causative factors, further infectious diseases and malnutrition might contribute to the high prevalence overall [61]. In patients with CKD, reduced GFR is a known risk factor to develop anaemia, especially below GFR ≤ 45 ml/min/1.73m^2^. In our population, only 10 out of 119 CKD cases have had an eGFR equal to or below 45ml/min/1.73m^2^. Overall, 90.5% of patients with CKD and anaemia had an eGFR >45 ml/min/1.73m^2^ and only 9.5% an eGFR ≤ 45ml/min/1.73m^2^ and importantly, the rate of patients with anaemia was equally distributed between cases with eGFR ≥ 60 (37.8%), eGFR 59–46 (58.3%), and eGFR ≤ 45 ml/min/1.73m^2^ (57.1%) (p = 0.293). These data suggest that anaemia is not explainable by CKD and reduced eGFR in all cases. Further investigations are needed to explore the question, whether anaemia is only a surrogate for chronic disorders, which in turn are risk factors for CKD or whether there is a role of anaemia as an independent causative factor for CKD.

Importantly, we performed our study in an outpatient clinic (OPC) clientele in a semirural area in Tanzania. Arogundade et al. suggested that a practical approach and a first step for prevention programs in SSA would be to engage in a general screening of all inhabitants attending hospitals for any reason [14]. This approach has the advantage that an access to the people is possible without additional effort and costs. However, one could expect that the estimation of CKD prevalence might be higher in an OPC-based compared to a community based setting [20–22]. Further, based on (patho)-physiological adaption mechanism in acute ill patients, alterations in GFR and albuminuria might be transient phenomenons and misclassified as CKD [62–64]. For this reason, we excluded patients with signs of acute systemic infection or UTI from CKD prevalence estimates. Compared to the estimated CKD prevalence of 13.6% the prevalence found in the excluded group would have been around 18%. Interestingly, all these patients had elevated albuminuria levels, but none of them had an impaired kidney function below an eGFR of 60ml/min/1.73m^2^. The absence of significant impaired kidney function in the excluded sub-population, indicates that the study population overall consisted of not seriously acute ill patients. Therefore, selection bias by including acute ill people with AKI and by miss-classifying those as CKD cases should be low.

Epidemiological studies in diverse populations have shown a strong association between the degree of albuminuria and the kidney outcome independently of the eGFR [34, 65–67]. Consistently, the KDIGO 2012 CKD classification is based on eGFR and albuminuria [28]. The recommended measurement is ACR from spot urine [28, 67, 68]. Compared to other studies in SSA the implementation of ACR measurements in all patients is a clear strength of our study. To the best of our knowledge quantitative assessment of albuminuria was not yet included in cross-sectional surveys investigating CKD in SSA [8, 20–22, 25, 28, 59]. In our study, 79% (94/119) of all CKD cases were classified based exclusively on elevated albuminuria levels without alterations in eGFR. Without testing for albuminuria the CKD prevalence estimate would have been only 2.8% (n = 25) compared to the calculated 13.6% (n = 119). Recently, two other studies were carried out investigating CKD prevalence in community based Tanzanian populations [21, 22]. Peck et al published prevalence rates between 2.3% to 7.5% based on eGFR solely [21]. Stanifer et al reported a CKD prevalence rate of 7% (95% CI; 3.8–12.3%) [22]. In this study, urine dipstick tests were performed to detect albuminuria [22]. In a recent survey from Uganda the estimated CKD prevalence was 9.8% using CKD-EPI formula and dipstick tests, but would have been only 0.2% without dipstick measurements [20]. Therefore, neglecting albuminuria leads to substantial errors in calculation of CKD prevalence rates and emphasizes the need of screening for albuminuria with reliable assays to predict prevalence rates most precisely [69, 70].

Epidemiological studies also differ in the application of different formula to calculate eGFR [20–22]. The CKD-EPI equation is recommended for population screening with unknown CKD status and is considered to be the most accurate eGFR in African adults [16, 17, 28, 33, 67]. In spite the fact, that the CKD-EPI equation leads to a lower estimated prevalence of CKD compared to the MDRD study equation, the CKD prevalence estimates in our study were equal or even higher compared to other studies in SSA [4, 8, 20–22].

### Limitations

In our study, we face several limitations. The estimated CKD prevalence data we are reporting are based on a single-point measurements of ACR and serum-creatinine. Therefore, prevalence of reduced eGFR and albuminuria might be overestimated. To the best of our knowledge there are no CKD prevalence studies in SSA without limitation of a single-point measurement. However, in our study the exclusion of patients with possible acute infection/inflammation and consecutively possible AKI and the exclusion of patients with suspected UTI and menstruation which could alter albuminuria should minimize selection bias by miss-classifying those cases as CKD cases. Nevertheless, there are multiple causes of AKI in the absence of acute infection/inflammation such as the ingestion of herbal remedies. Therefore, overestimation of our prevalence estimates cannot be fully excluded.

Further, due the fact of single-point measurements and a cross-sectional design, our data do not allow to draw any conclusion about causality and effect of the analysed variables in regard of impaired kidney function or albuminuria. A major limitation is that office BP was assessed by a single measurement only. Therefore, conclusions about BP have to be cautiously drawn, and statements about BP categories/stages and prevalence rates of hypertension are not possible. A further limitation of our study is that certain conditions, especially the history of infectious diseases, were assessed mainly based on a history of recall and not on objectifiable data. Therefore, in future studies further assessments and longitudinal observations have to be carried out to determine their role in kidney disease.

Finally, there was no screen log implemented to record systematically the rate of participants in the study. However, due to the setting of the study, the response rate was high and almost all patients were willing to participate in the study. The reasons for the high response rate were, that the frame of the regular consultation hour was not disturbed, but the quality increased by a standardized questionnaire, the investigation of basic vital parameters (height, weight, blood pressure) and by additional blood and urine examinations without any expenditure for the participants. Further, because of the nature of the study with a cross-sectional design, there were no follow-up visits or other inconveniences for the participants.

### Conclusion

Our study conducted in a semi-rural population extends the existing epidemiological data set for CKD and provides further evidence for high CKD prevalence in SSA. Particularly, our findings support the hypothesis that people living in SSA are affected by a “double burden” of non-communicable and endemic infectious disorders which both might contribute to a steady rise of CKD in SSA [3, 43]. In a next step, we need high quality longitudinal epidemiological data and a better understanding of the pathophysiological role and the impact of neglected diseases on kidney health [20, 21].
